# Abstract of the Proceedings of the Illinois State Medical Society, at the Annual Meeting, May 21-22, 1872

**Published:** 1872-06-01

**Authors:** 


### Society Reports.

### ABSTRACT OF THE PROCEEDINGS OF THE ILLINOIS STATE MEDICAL SOCIETY, AT THE ANNUAL MEETING, MAY 21-22, 1872.

(*From Notes by the Editor.*)

The members of the Society assembled in the hall at Rock Island on Tuesday morning, May 21st, and were called to order by the President, Dr. J. O. Hamilton, of Jeseyville, at 10.30 o'clock a.m.

After prayer by the Chaplain, and the report of the Committee of Arrangements on the registration of members, Dr. Gregg, Chairman of that Committee, welcomed the members to the hospitalities of the city, in a very appropriate address.

The list of Standing and Special Committees was then called; and out of five Standing Committees only one, that on Drugs and Medicines, was prepared with a regular report. Of the nineteen Committees on special subjects, appointed at the previous annual meeting, five only announced their readiness to report. Volunteer papers were offered by Drs. E. L. Holmes, A. L. McArthur, and F. C. Hotz.

Dr. T. D. Washburn, one of the Committee on Practical Medicine, presented a paper which he had designed as part of the report to be made by the Chairman of that Committee. Although so small a proportion of the Committees reported, yet the number of reports and papers read was sufficient to occupy the attention of the Society closely for two days.

On motion of Dr. D. W. Young, a resolution was adopted declaring that no reports or papers should be referred to the Committee on Publication, except such as are written out in full and ready to hand to the Secretary when read to the Society.

Dr. S. P. Breed, of Princeton, read a lengthy report on the subject of "Organic Dynamics," referring more particularly to the cerebro-spinal and organic systems of nerves.

He claimed that all dynamic force originated in the organic cells; that the cells of vegetable organization receive and correlate the caloric, light, and electricity into organic force; and that the nerve cells derive their force from the vegetable cells. He likened the nerve cells to galvanic batteries, and the nervous cords to the conducting wires. It was stated that every difference of function implied a difference in structure; that no two cells were alike: or that the cells of the brain of no two individuals of the same family or species were alike; and consequently they could not think alike.

It was claimed by the writer that cells, while developing, absorbed force, and gave it out only in the process of dissolution. This highest type of cell development was the *ideational* cells of the cerebrum, which in their dissolution evolve the phenomena of mind. The report closed with the detail of some cases of disease intended by the writer as illustrations of his previously developed theories. The report was referred to the Committee of Publication without discussion.

Dr. J. H. Hollister, of Chicago, Chairman of the Committee on Drugs and Medicines, read his report, which was one of much practical importance. He referred briefly to the importance of pharmacy, and its recent progress, and then proceeded to show the nature and extent of some of the more important adulterations and sophistications of drugs. He also presented, as a part of his report, a short paper by Dr. A. Niles, of Quincy, a member of the Committee on new Remedies. The report fully sustained the fact, already well known, that many of the most important articles of the materia medica are so extensively adulterated, as kept in many of the drug stores in all parts of the country, that great care is required on the part of the practitioner to avoid impositions and consequent disappointment in the effects of his remedies. The principal new remedies referred to were the bromal hydrate as a substitute for chloral hydrate, in cases requiring prolonged anæsthesia; the bromides of quinine and morphine in the treatment of neuralgia and diabetes; phosphide of iron in cases requiring more phosphorus in the system; and pepsin as obtained by macerating fresh rennet in glycerine. In the discussion which followed the reading of the report, Dr. E. Andrews, of Chicago, spoke of the great importance of drug adulterations, and related some instances of importance coming under his own observation in Chicago. He suggested, as the most efficient remedy, the active examination of the more important drugs in every market, and the prompt exposure of all clearly ascertained adulterations, through the columns of the public press.

Dr. D. Prince, of Jacksonville, made some inquiries in regard to the bromo-chloralum, and stated that he had used it in a number of cases, and thought it entirely useless.

Dr. E. R. Travers had used the bromochloralum in a case of cancer, to correct the fetid character of the discharge, but found it worthless.

Dr. D. W. Young, of Aurora, said, in reference to the subject of adulterated and worthless drugs, that physicians might do much to remedy the evil by greater care and discrimination in the bestowal of their patronage. He suggested that physicians in all the more populous towns and cities should club together and procure the establishment of drug stores exclusively for physicians' prescriptions, in which nothing but drugs selected specially for filling prescriptions should be kept.

The report of Dr. Hollister was referred to the Committee of Publication.

The report of Dr. C. W. Earle, of Chicago, Special Committee, on the recent advancements in physiology, was presented by the Secretary, the author not being able to attend the meeting. The report was received, and referred to the Committee of Publication.

Dr. E. L. Holmes, of Chicago, presented a short volunteer paper on the "Diagnosis and Treatment of Iritis." He stated that the existence of a certain grade of iritis was often overlooked, from the absence of well-marked symptoms. In such cases, he said, a drop of solution of atropia in the eye, causing dilatation of the pupil, would cause the irregular margin of the dilated pupil to be easily recognized. He recommended iridectomy as an important means for relieving inflammation and abscess of the cornea. He alluded to the use of calomel as a local application in chronic inflammation, involving the conjunctiva; and while admitting its beneficial effects, cautioned against its application while the patient was, at the same time, taking the iodide of potassa internally; the latter being capable of impregnating the tears, and entering into chemical combination with the mercurial, and thereby causing severe pain, with increase of the inflammation. A case of aneurism, or pulsating tumor of the orbit, was referred to, in which the use of veratrum viride was tried, which materially lessened the tumor, but failed to effect a cure. It was then injected with a solution of per-sulphate of iron, with entire success. He also related two cases of acute mania following operations for cataract. Both cases commenced about three days after the operation, and continued several days. The question of chief interest was whether the mental derangement in such cases was due to the atropia used locally for dilating the pupil?

Dr. D. Prince, of Jacksonville, stated that a case had occurred under his observation in which a single drop of the atropia, applied to the eye for the purpose of making an examination, was followed by insanity of three days duration.

Dr. J. H. Hollister related a case of chronic conjunctivitis, which was rapidly cured by the application of an ointment of the biniodide of mercury to the outside of the eyelids.

EVENING SESSION.

The meeting was called to order at 7.30 p.m., the President, Dr. J. O. Hamilton, in the chair.

The Secretary announced the names of the Committee on Nominations.

Dr. F. C. Hotz, of Chicago, presented and read a paper on the use of sulphate of quinia as a local application in the treatment of vascular cornea, pannus, and granular lids, including the detail of several cases illustrating the effects of the remedy.

He stated that dry quinine dusted on the conjunctival surface of the lids twice each day, pretty uniformly resulted in a rapid improvement of the condition of the cornea; but that it had no effect on the granulation of the lids. The first applications were generally productive of moderate pain, but which became less at each repetition. In only one case did he find the irritation such as to require a discontinuance of the application. It was well borne in many cases of conjunctivo-corneitis, in which the application of sulphate of copper could not be tolerated. The cases most promptly and fully relieved by the local application of the quinine, were those of pannus, and moderate inflammation of the cornea, without any accompanying granulations on the lids.

Dr. D. Prince remarked that quinine had been used as a local application to almost every variety of ulcer. He thought it acted as an astringent, diminishing the caliber of the inflamed vessels.

Dr. E. L. Holmes stated that his trials of the use of quinine as a local application had resulted generally in improving the condition of the cornea, but in no case had had any effect on granulated lids.

Dr. Blackman regarded the internal use of quinine very beneficial in most cases of chronic conjunctivo-corneitic inflammation.

Dr. Crawford expressed the same opinion concerning the importance of quinine as an internal remedy.

Dr. E. P. Cook thought the internal use of quinine and tonics chiefly beneficial in the ill-fed class of patients.

Dr. D. W. Young stated that he had used quinine as a local application in the treatment of gleet, with more benefit than any other remedy. He generally used a solution of 8 grains to the ounce of water.

After some remarks by Dr. N. S. Davis on the different varieties of conjunctival and corneitic inflammations, and the variations of treatment required, the paper of Dr. Hotz was referred to the Committee of Publication.

Dr. E. R. Travers, of Amboy, Special Committee on Dystosia, read his report, which was chiefly devoted to the subject of "Labors rendered difficult from fault in the soft parts, as distinguished from those rendered difficult either by defects or deformities of the bones of the pelvis, or in the fœtus.

Rigidity of the os-uteri was represented as one of the most frequent causes of dystosia. To produce relaxation, and thereby facilitate the progress of labor, he recommended nauseating doses of tartrate of antimony and potassa; and in cases of unusual persistence, the incising of the os, either laterally or anteriorly.

He related a case in which there was both unusual rigidity and hyperæsthesia of the os, and which resisted the influence of tartar emetic, chloroform, and other remedies, and which in its progress became complicated with convulsions. The os was then freely incised, and an attempt made to deliver with the forceps; but failing in this he resorted to craniotomy.

In commenting on this paper, Dr. Prince stated that tartar emetic as a remedy for relaxing the os in labor, had been abundantly tried and very generally abandoned as liable to induce troublesome vomiting and excessive prostration. He thought chloroform a far more efficient and and less objectionable remedy.

He related an interesting case of pregnancy in a woman who had a great degree of hypertrophy and induration of the os and cervex uteri, from long-continued prolapsus. She had suffered much delay and injury in a previous confinement; and knowing from the condition of the tissues that the same suffering would result again unless averted by some interference, it was determined to attempt a full control of the subsequent labor. About two weeks before the expected labor, proper measures were taken to bring on labor prematurely by dilating the os. This succeeded in soon inducing regular and efficient labor pains, with firm pressure of the head of the child on the unyielding os. A free lateral incision was now made through the dense structure of the os and cervex, and the labor completed with entire safety to both mother and child. Dr. Prince thought incising the os comparatively free from danger, but necessary in only a very small number of cases.

Dr. D. W. Young thought neither incisions nor premature labor should be practiced except in extreme cases.

Dr. T. D. Fitch had never had occasion to incise the os, or to give tartar emetic in labor. He thought, when rigidity of the os existed, or the occurrence of convulsions made speedy delivery necessary, chloroform much the best remedy. If it fails, incision of the rigid os might be necessary, and should always be lateral. He said more attention should be given, in retarded and difficult labors, to malpositions of the os. It was often found pressed backward into the concavity of the sacrum, or anteriorly over the pubis; and required judicious manipulation to place it in the right direction compared with the axis of the pelvis.

Dr. P. Gregg stated that he had attended over 1800 cases of labor, but had never had occasion to incise the os. He thought there was at the present time too much disposition to cut and pull the womb about. He asked what would prevent the incisions from being torn up further during the strong contractions of the uterus in expelling the child?

Dr. D. Prince replied that the circular fibres of the uterus at the full term of pregnancy were very strong, and did not lacerate. But practically the incisions made for relieving rigidity of the os, did not reach the body of the uterus, but were limited to the tissues belonging to the os and cervex.

Dr. H. VanDusen, of Mineral Point, delegate from the Wisconsin State Medical Society, spoke of the value of opium judiciously used, to procure rest first, and subsequent relaxation, in difficult labors.

After some further remarks from Dr. Travers, the Society adjourned to 8.30 a.m.

WEDNESDAY MORNING SESSION.

The meeting was called to order at 9 a.m., the President, Dr. J. O. Hamilton, in the chair.

Dr. E. Powell, of Chicago, Special Committee on the Transplantation of Cuticle, read his report.

From the trials he had made, he regarded it as quite certain that many large ulcers can be made to heal or cicatrize by the aid of transplantation, which would not heal without it; and others can be made to cicatrize much faster. It was also stated that the new skin formed by this process was less sensitive and more firm than when cicatrization takes place without it. He recommends first cleansing the surface of the ulcer with a solution of carbolic acid; then strip out a bit of healthy skin or cuticle, cut it into small pieces not larger than millet seeds; superficially incise the surface of the ulcer, and sprinkle the bits of cuticle over it, and cover it with adhesive plaster. He leaves it undisturbed for three or four days. On removing the plaster at the end of that time, little red points and islands of new skin will be seen on the surface of the ulcer, which in a few days more are found to be so many points of increasing cicatrization.

Dr. E. Andrews stated that he had seen some quite large pieces of cuticle adhere when applied in the manner described by Dr. Powell. He said in one case the integument used was kept in a vial two hours after it had been removed before it was sprinkled in the ulcer, and yet it succeeded well. He also referred to a case in which a large gap in the substance of a bone refused to heal for a long time. He paved the bottom of the ulcer with bits of skin one-eighth of an inch in diameter, with entire success.

Dr. E. Powell stated that he had several times tried the method of Dr. Hodge, which was to sprinkle on the surface of the ulcer the mere scarf-skin or cuticle scraped from the surface, but had never made it succeed. This report was referred to the Committee of Publication.

Dr. P. Gregg reported verbally a case of vesico-vaginal fistula, in which there was very extensive loss of substance, consequent on injury to the soft parts during a tedious labor. This first operation for closing the opening succeeded in reducing its size about one-half. The second operation resulted in the closure of all but a small aperture; and the patient could retain a small quantity of urine at a time, and pass it through the urethra. In these operations it had been found necessary to so include the neck of the uterus that the os was turned into the bladder. As the general health of the woman was bad, he deferred any further operative procedure for a few weeks, to allow time for improvement.

Little more than two months passed, and he was called in haste to the patient; and very much to his surprise found she had had a miscarriage, having expelled a fœtus of six or eight weeks process. That impregnation should have taken place through a small aperture in the vaginal wall, and still more, that such aperture should have dilated so as to allow the fœtus to escape through it, he regarded as very remarkable.

After her recovery from the abortion, Dr. Gregg again operated on the fistula, and succeeded in effecting an entire closure, leaving the os uteri within the bladder. She recovered good health, and ever after menstruated regularly through the urethra.

Dr. S. K. Crawford, Special Committee on Criminal Abortions, read his report. Nothing new was developed concerning the subject; and the report was referred to the Committee of Publication.

Dr. T. D. Washburn, of Hillsboro, a member of the Standing Committee on Practical Medicine, presented and read the paper that he had designed to constitute a part of the report of the Committee. It related, chiefly, to the topography and diseases of Montgomery County; and was referred to the Committee of Publication.

Dr. A. L. McArthur, of Rockford, read a volunteer paper on the "Formation of Bone." The paper evinced much thought and research, referring to most of the experiments and observations made in this country concerning the reparation and reproduction of bone.

His conclusions were, that bone is capable of being reproduced from periosteum, from the medullary substance or marrow, from the cancellated structure, and even from the inherent formative powers of the system, without the presence of either periosteum or any part of bone. The paper embraced a brief history of four cases illustrating the subject under discussion.

A discussion followed the reading of the paper, participated in by Drs. Hamilton, Truesdale, Young, VanDusen, Crawford, Prince, and Fitch. The cases related were interesting, but no new items of practical value were developed. The paper of Dr. McArthur was referred to the Committee of Publication.

